# Vasorelaxant and hypotensive effects of an ethanolic extract of *Nymphaea pubescens* and its main compound quercetin 3-methyl ether 3′-O-β-xylopyranoside

**DOI:** 10.3389/fphar.2024.1379752

**Published:** 2024-03-21

**Authors:** Teerapap Panklai, Kornkanok Ingkaninan, Krongkarn Chootip, Prapapan Temkitthawon, Nungruthai Suphrom, Maude Tournier-Nappey, Corine Girard, Céline Demougeot, Perle Totoson

**Affiliations:** ^1^ Center of Excellence in Cannabis Research, Faculty of Pharmaceutical Sciences and Center of Excellence for Innovation in Chemistry, Naresuan University, Phitsanulok, Thailand; ^2^ Université de Franche-Comté, EFS, INSERM, UMR RIGHT, Besançon, France; ^3^ Department of Physiology, Faculty of Medical Science and Center of Excellence for Innovation in Chemistry, Naresuan University, Phitsanulok, Thailand; ^4^ Center of Excellence in Biomaterials, Faculty of Science and Center of Excellence for Innovation in Chemistry, Naresuan University, Phitsanulok, Thailand

**Keywords:** *Nymphaea pubescens*, vasorelaxation, mesenteric artery, hypotensive effect, compound

## Abstract

**Aim:** *Nymphaea* plants were traditionally used to treat diseases associated with endothelial dysfunction. The present study investigated the effects of an ethanolic extract of *Nymphaea pubescens* Willd. (commonly named water lily, WL) and its main compound **1** (quercetin 3-methyl ether 3′-O-*β*-xylopyranoside) on vascular function in rats.

**Materials and methods:** The vasorelaxant effects of the WL extract and its main compound **1** and their underlying mechanisms of action were evaluated on isolated mesenteric arteries from Wistar rats. Blood pressure and heart rate were measured in anesthetized rats after infusion (i.v) of vehicle, WL extract, and compound **1** (at 0.01, 0.025, 0.05, 0.1, 0.5, and 1 mg/kg). Nifedipine was used as a positive control.

**Results:** Both WL extract and compound **1** induced vasorelaxant effects (with EC_50_ of 0.08 ± 0.01 mg/mL and 42.8 ± 6.3 µM, respectively) that were reduced by endothelium removal. A significant decrease in these relaxations was observed with L-NAME but not with apamin–charybdotoxin or indomethacin. In the endothelium-denuded condition, WL extract-induced relaxation was enhanced by 4-aminopyridine and glibenclamide, while iberiotoxin and ODQ (1H-[1,2,4]oxadiazolo[4,3-a]quinoxaline-1-one) had no effect. In contrast, compound **1**-induced relaxation was not changed by any of these inhibitors. Both WL extract and compound **1** enhanced sodium nitroprusside-induced relaxation and inhibited receptor-operated Ca^2+^ channels. Only the WL extract was able to reduce PE-induced contraction (*p* < 0.001). As compared to the vehicle, the infusion of WL extract and compound **1** lowered systolic and diastolic blood pressure. Interestingly, the hypotensive effect of the compound was similar to that of nifedipine. The rebound tachycardia found at the highest dose of nifedipine was not observed with the WL extract or compound **1** (*p* < 0.05).

**Conclusion and discussion:** Our study demonstrated a vasorelaxant effect of the WL extract and its main compound quercetin 3-methyl ether 3′-O-*β*-xylopyranoside, relying on the potentiation of the NO-cGMP pathway and calcium inhibitory effects. These vasorelaxant effects were associated with a potent hypotensive effect, providing pharmacological evidence for the traditional use of this plant.

## 1 Introduction

The family Nymphaeaceae includes aquatic plants commonly named water lilies (WLs) and is classified into six genera, namely, *Barclaya*, *Euryale*, *Ondinea, Victoria, Nuphar*, and *Nymphaea*. *Nymphaea* is the most diverse and widespread, almost worldwide, and comprises approximately 45–50 species (Selvakumari et al., 2016). In many rural areas, young flowers and peduncles of *Nymphaea* are consumed fresh, cooked as vegetables, or used for the treatment of diseases such as erectile dysfunction or cardiometabolic diseases. Thus, the flowers of *Nymphaea pubescens* Willd. are widely used in many Asian countries as enhancers of erection (La-ongsri et al., 2009). In addition, ethnopharmacology studies demonstrated that extracts from *N. pubescens* petals had anti-diabetic, hypolipidemic, and antioxidant properties (Sathasivampillai and Rajamanoharan, 2021; Pokhrel et al., 2022). As the common hallmark of these diseases is the presence of endothelial dysfunction (Musicki et al., 2015; Konstantinovsky A et al., 2019; Evans CE et al., 2021), the above data suggest that extracts from *N. pubescens* petals have direct endothelial actions. Consistent with this hypothesis, previous studies using extracts from *Nymphaea lotus* Linn reported their capacity to enhance nitric oxide (NO) production from the endothelium of the aorta and corpora cavernosa (Mireille et al., 2016; Kameni et al., 2019). However, whether *N. pubescens* extract induces such a favorable effect on endothelial NO production has never been investigated. Of note, our previous work revealed that the main compound of an ethanolic extract of flowers from *N. pubescens* is a quercetin derivative, quercetin 3-methyl ether 3′-O-*β*-xylopyranoside (Panklai et al., 2022; Panklai et al., 2023). As quercetin is well-known to induce vasorelaxant effects through NO-dependent effects (Maaliki et al., 2019; Ciumărnean et al., 2020; Chen and Zhang, 2021; Grosso et al., 2022), this main compound might contribute to a positive endothelial effect in *N. pubescens*.

In the present study, the effects of an ethanolic extract of *N. pubescens* petals and its main compound (quercetin 3-methyl ether 3′-O-*β*-xylopyranoside, named compound **1**) were studied in an isolated rat mesenteric artery, with a focus on their endothelium-dependent and endothelium-independent mechanisms. To assess whether the results obtained *ex vivo* translated into a pharmacological effect *in vivo*, their acute hypotensive effect was determined in anesthetized rats.

## 2 Materials and methods

### 2.1 Plant names and parts used

The ptals of *N. pubescens* (WL) were collected from Phitsanulok at the Faculty of Pharmaceutical Science of Naresuan University, Thailand. The voucher specimen was identified by Assistant Professor Dr. Pranee Nangngam, Department of Biology, and kept at the Faculty of Sciences, Naresuan University, Phitsanulok, under No. 004664. The petals were first dried in a hot air oven at 55°C for 2 days. This dried material (182 g) was ground into powder and macerated with 95% ethanol (1.5 L) for 3 days (two times). Then, it was filtered and evaporated until dryness to give a yield of 32.33% (w/w) of crude ethanolic extract. In brief, compound **1** was isolated and purified using a solid phase extraction (SPE) mini-column, Strata C18-E (55 μm, 70 A), and a preparative PLC (Gilson PLC 2020) fitted with a Kinetex EVO Reverse-Phase C18 Column (250 × 21.2 mm, 5 µm). Spectroscopic analysis was used to elucidate the structure of the isolated compound. The purity of compound **1** was greater than 98%, as measured by HPLC. The WL extract and compound **1** were stored at −20 °C until used.

### 2.2 Animal studies

Male Wistar rats (8–12 weeks old) were purchased from Janvier (Le Genest-Saint-Isle, France). Animals were kept at 22°C ± 1°C, under a 12–12 h light/dark cycle, with free access to drinking water and food pellets. The experimental design of the study was approved by the local committees for ethics in animal experimentation at the University of Franche-Comté (Besançon, France) under the number 2019/003-PT/5PR. All the investigation conforms to the Guiding Principles for Research Involving Animals: ARRIVE animal research.

### 2.3 Preparation of mesenteric arteries

The rats were anesthetized by intraperitoneal administration of sodium pentobarbital (60 mg/kg) and exsanguinated. Then, we excised the second-order branches of mesenteric arteries (MAs), cleaned them of connective tissue, and cut them into rings ∼2 mm in length. The MA rings were loaded into organ chambers containing 6 mL of Krebs buffer (composition: 118 mM NaCl, 4.7 mM KCl, 1.2 mM KH_2_PO_4_, 1.2 mM MgSO_4_, 2.5 mM CaCl_2_, 25 mM NaHCO_3_, and 12 mM glucose) at 37°C and continuously aerated with 95% O_2_ and 5% CO_2_. The MA rings were threaded on two stainless steel wires of 40 µm diameter. The contractile response (isometric force in mN) was measured by a force transducer connected to a multi-myograph system (Model 610 M v.2.2, DMT A/S, Denmark) and coupled to a data acquisition system: ChartTM Ver.7 software (ADInstruments, France). Resting tension was fixed for an initial equilibration period by stretching to their optimal lumen diameter. This optimal lumen diameter was chosen by setting the internal circumference to 90% of what the vessels would have if they were exposed to a transmural pressure of 100 mmHg. The MA rings were equilibrated for 15 min with normal Krebs. Then, they were routinely challenged with a 100 mM KCl solution to measure the vessel viability. The presence of functional endothelial cells was verified by pre-contracting with phenylephrine (PE, 10^−5^ M) and adding acetylcholine (ACh, 10^−5^ M) to induce more than 80% relaxation. In some rings, the endothelium was mechanically removed by gently rubbing inside the vessel with small mouse whiskers (Wisutthathum et al., 2018). The relaxation response to ACh (10^–5^ M) of less than 10% attested to the completeness of this endothelial denudation.

#### 2.3.1 Study of the vasorelaxant effect of WL extract and compound 1

Endothelium-intact (E+) MA rings and endothelium-denuded (E−) MA rings were sub-maximally pre-contracted with PE (10^−5^ M), and then the response to cumulative concentrations of WL extract (10^−5^-1 mg/mL) or compound **1** (10^−7^–10^−4^ M) was determined to obtain concentration–response curves. The relaxation effect was calculated as the percentage of the contraction in response to PE. The effect of the vehicle (DMSO 0.09% for the WL extract and 0.1% for the compound **1**) was evaluated under the same conditions.

#### 2.3.2 Role of endothelium-dependent pathways

The role of three endothelium-dependent pathways consisting of i) nitric oxide synthase (NOS), ii) cyclooxygenase (COX), and iii) endothelium-derived hyperpolarizing factor (EDHF) in the vasorelaxant activities of WL extract and compound **1** was investigated. For this purpose, the vasorelaxant effects of WL extract or compound **1** were studied in (E+) MA rings in the presence of N^G^-nitro-L-arginine methyl ester (L-NAME, 10^−4^ M); an NOS inhibitor, indomethacin (10^−5^ M); a COX inhibitor or apamin (10^−7^ M) and charybdotoxin (10^−7^ M); and small- and intermediate-conductance Ca^2+^-activated K^+^ channel blockers (SK_Ca_ and IK_Ca_) (Wisutthathum et al., 2018).

#### 2.3.3 Role of vascular smooth muscle K^+^ channels

To investigate the contribution of three types of K^+^ channels to the relaxant effect of WL extract and compound **1**, (E−) MA rings were pre-incubated with i) glibenclamide (10^−5^ M), an ATP-sensitive potassium channel (K_ATP_) blocker; ii) 4-aminopyridine (4-AP, 10^−3^ M), a voltage-gated potassium channel (K_V_) blocker; or iii) iberiotoxin (10^−7^ M), a large-conductance Ca^2+^-activated K^+^ channel (K_Ca_) blocker for 30 min. Then, PE (10^−5^ M) was added, and the cumulative concentration effects of WL extract or compound **1** were studied (Wisutthathum et al., 2018).

#### 2.3.4 Role of the soluble guanylyl cyclase and cyclic guanosine monophosphate pathway

We investigated the involvement of the soluble guanylyl cyclase (sGC)/cyclic guanosine monophosphate (cGMP) pathway in the relaxant effects induced by WL extract and compound **1.** To explore whether WL extract and compound **1** might modulate cGMP levels, the (E−) MA rings were incubated for 10 min with WL extract (at EC_50_ of 0.25 mg/mL), compound **1** (at EC_50_ of 100 µM), or the vehicle (0.03% for the WL extract and 0.1% for the **1**). Then, cumulative concentration effects (10^−11^–10^−4^ M) of sodium nitroprusside (SNP), a NO donor, were studied in 100 mM KCl-preconstricted rings (for the WL extract) or PE (10^−5^ M)-preconstricted rings (for compound **1**). Second, to determine whether a direct activation of sGC was involved in the WL extract- or compound **1-**induced relaxations, (E−) MA rings were incubated for 30 min with 1H-[1,2,4]oxadiazolo[4,3-a]quinoxaline-1-one (ODQ, 10^−5^ M), a selective inhibitor of sGC, before adding 10^−5^ M of PE and subsequent cumulative concentrations of WL extract or compound **1**.

#### 2.3.5 Possible action of WL extract or compound 1 on extracellular Ca^2+^-induced contraction and sarcoplasmic reticulum Ca^2+^ release

To investigate the role of extracellular calcium (Ca^2+^) influx in the WL extract- or compound **1**-induced relaxations, (E−) MA were incubated with a Ca^2+^-free Krebs’ solution containing methylene glycol-bis (2-aminoethylether)-N,N,N′,N′-tetraacetic acid (EGTA, 2 mM) for 40 min. Then, PE (10^−5^ M) was added, and rings were washed with the Ca^2+^-free Krebs’ solution for 30 min (washed three times every 10 min) to deplete intracellular Ca^2+^ stores from the sarcoplasmic reticulum (SR). Then, MAs were incubated at EC_50_ of the WL extract (0.25 mg/mL), compound **1** (100 µM), or the vehicle, respectively, for 10 min, before adding PE (10^−5^ M) or 80 mM KCl for the opening of receptor-operated Ca^2+^ channels (ROCCs) or voltage-operated Ca^2+^ channels (VOCCs). CaCl_2_ (10^−2^ M) was added to induce a contractile response.

To assess the effect of WL extract and compound **1** on the intracellular calcium (Ca^2+^) release, (E−) MA rings were incubated with a Krebs’ solution for 40 min, then incubated with the L-type voltage-dependent Ca^2+^ channel inhibitor (verapamil, 10^−7^ M) for 30 min. Then, MA rings were incubated at EC_50_ of the WL extract (0.25 mg/mL), compound **1** (100 µM), or the vehicle for 15 min. After that, PE (10^−5^ M) was added to the bath, and the transient contraction was determined to estimate the amount of Ca^2+^ release from the sarcoplasmic reticulum.

#### 2.3.6 Role of α_1_ receptor in WL extract and its main compound-induced relaxations

For this purpose, (E−) MA rings were incubated at EC_50_ of the WL extract (0.25 mg/mL, compound **1** (100 µM), or the vehicle for 15 min before cumulative vasocontraction was performed with PE (10^−10^–10^−4^ M). The results were obtained in percentage contraction by comparison of the maximum contraction of PE (10^−5^ M) without treatment (Paracha et al., 2019).

### 2.4 Blood pressure and heart rate measurements

To assess whether the direct *in vitro* vascular effect of WL extract and compound **1** on resistance vessels translated into an *in vivo* effect, we studied the acute hypotensive effect of WL extract and compound **1** in anesthetized rats. Normotensive male Wistar rats were anesthetized with pentobarbital (60 mg/kg, i.p.); then, the left carotid artery was catheterized using a polyethylene tube (0.279 mm i.d. × 0.609 mm o.d.) filled with heparinized saline(50 units/mL saline) and connected to a pressure transducer (model BP-100 Blood Pressure Transducer, iWorx Systems, Inc., Dover, NH, United States). The output pressure was recorded by using a bridge amplifier coupled to PowerLab^®^ Recording System and a Chart™ application program (Ver.6 ADInstruments, Castle Hill, NSW, Australia). In addition, 15 min of stabilization period was applied, and then systolic blood pressure (SBP), diastolic blood pressure (DBP), and heart rate (HR) were recorded before and during intravenous injection (at 1 mL/min) of vehicle (a saline solution containing 5% DMSO, 1 mL/kg) or cumulative and increasing doses (0.01, 0.025, 0.05, 0.1, 0.5, and 1 mg/kg) of WL extract, compound **1,** or nifedipine. Each drug was administered to a different animal.

### 2.5 Drugs

4-AP, ACh, apamin, EGTA, glibenclamide, iberiotoxin, indomethacin, L-NAME, nifedipine, ODQ, PE, SNP, and verapamil were purchased from Sigma ChemicalCompany (St. Louis, MO, U.S.A.). Charybdotoxin was obtained from Enzo Life Sciences Company (France), and DMSO was obtained from VWR International Ltd. (Prolabo Chemicals, United Kingdom). All substances were dissolved in distilled water except 4-AP, compound **1,** glibenclamide, nifedipine, and WL extract, which were dissolved in DMSO and indomethacin in 0.5% w/v Na_2_CO_3_.

### 2.6 Statistical analysis

Values represent the means ± standard error of the mean (SEM). The WL extract- and compound **1**-induced vasorelaxations were calculated as the percentage of contraction to PE (10^−5^ M). The concentration of WL extract or compound **1** that induced 50% of the maximal relaxation (EC_50_) and maximal relaxation induced (E_max_) was determined by logit transformation of the normalized concentration–response curves using the 5.0 version of GraphPad Prism software. The concentration–response curves were compared using a two-way ANOVA for repeated measures, followed by a Bonferroni’s test. A comparison between two values was assessed using an unpaired Student’s t-test or Mann–Whitney *U* test when the data were not normally distributed. The *p*-value < 0.05 was considered to be significantly different.

## 3 Results

### 3.1 WL extract and its main compound induced vasorelaxant effect, relying on both endothelium-dependent and endothelium-independent mechanisms

**FIGURE 1:**
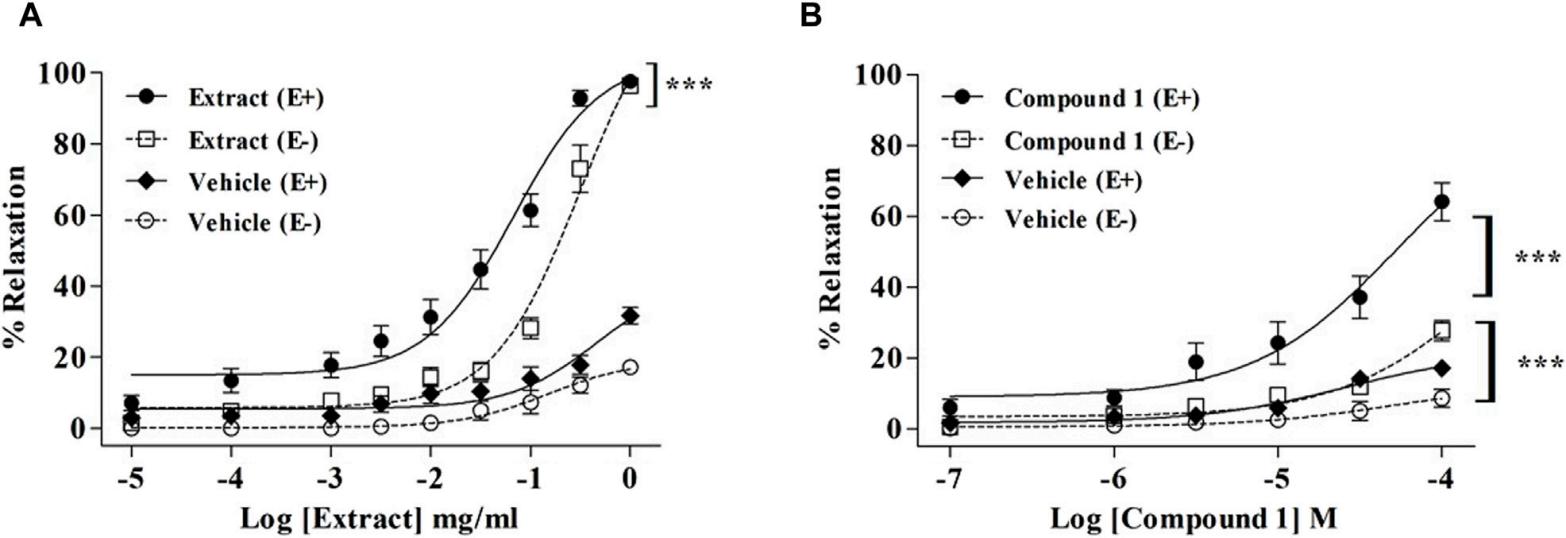
Relaxation of rat mesenteric artery rings pre-contracted with PE (10^−5^ M) and treated with accumulating concentrations of WL extract **(A)** or quercetin 3-methyl ether 3′-O-*β*-xylopyranoside **(1) (B)** in endothelium-intact (E+) and -denuded (E−). Relaxations are expressed as % contractions induced by PE. The values are presented as the means ± SEM (n = 5–11); ^***^ *p* < 0.001.

As compared to the vehicle, WL extract (E_max_ = 97.90 ± 1.17%; EC_50_ = 0.08 ± 0.01 mg/mL) and compound **1** (E_max_ = 64.14 ± 5.08%; EC_50_ = 42.76 ± 6.32 µM) induced a concentration-dependent vasorelaxation in (E+) MA rings (Figure 1; Table 1). The removal of the endothelium significantly decreased the relaxant effect in both WL extract and compound **1**, as confirmed by the increase in the EC_50_ values to 0.25 ± 0.03 mg/mL and 91.42 ± 18.45 µM, respectively (*p* < 0.001).

**TABLE 1:** EC_50_ and E_max_ of WL extract- or quercetin 3-methyl ether 3′-O-*β*-xylopyranoside **(1)**-induced relaxations in mesenteric artery rings in the absence or presence of various inhibitors.

	WL extract		
	EC_50_ (mg/mL)	E_max_ (%)	n
Endothelium intact (E+)			
Vehicle	-	31.66 ± 2.08	5
Extract	0.08 ± 0.01	97.90 ± 1.17	11
+ L-NAME	0.18 ± 0.02	95.85 ± 1.08	7
+ Indomethacin	0.04 ± 0.01	95.49 ± 1.03	7
+ Apamin + charybdotoxin	0.03 ± 0.01	97.69 ± 0.36	7
Endothelium denuded (E−)			
Vehicle	-	17.17 ± 1.27	5
Extract	0.25 ± 0.03***	96.43 ± 0.73	11
+4-AP	0.06 ± 0.02††	98.09 ± 0.66	7
+ Glibenclamide	0.14 ± 0.04	98.00 ± 0.56	7
+ Iberiotoxin	0.26 ± 0.05	95.01 ± 0.71	7
+ ODQ	0.14 ± 0.03	89.45 ± 4.60	7
	Quercetin 3-methyl ether 3′-O-β-xylopyranoside (1)		
	EC_50_ (µM)	E_max_ (%)	n
Endothelium intact (E+)			
Vehicle	-	17.10 ± 1.17	5
1	42.76 ± 6.32	64.14 ± 5.08	10
+ L-NAME	>100	43.16 ± 7.71	7
+ Indomethacin	38.67 ± 8.35	78.41 ± 7.14	7
+ Apamin + charybdotoxin	4.42 ± 0.78**	79.56 ± 3.59	7
Endothelium denuded (E−)			
Vehicle	-	8.58 ± 2.29	5
1	>100	27.71 ± 2.66***	10
+4-AP	>100	36.40 ± 7.80	6
+ Glibenclamide	>100	40.04 ± 7.67	6
+ Iberiotoxin	>100	19.08 ± 2.34	6
+ ODQ	>100	40.19 ± 7.95	7

Values are presented as the means ± SEM. EC_50_ indicates the concentration of WL extract or compound **1** giving half-maximal relaxation. E_max_ is the maximum response of MA and is expressed as a relaxation percentage of the PE-induced contraction.

**p < 0.01.

***p < 0.001 vs Extract (E+) or 1 (E+).

††*p* < 0.01 vs Extract (E−).

### 3.2 The endothelium-dependent vasodilation of WL extract and compound 1 is mainly NOS-dependent

**FIGURE 2:**
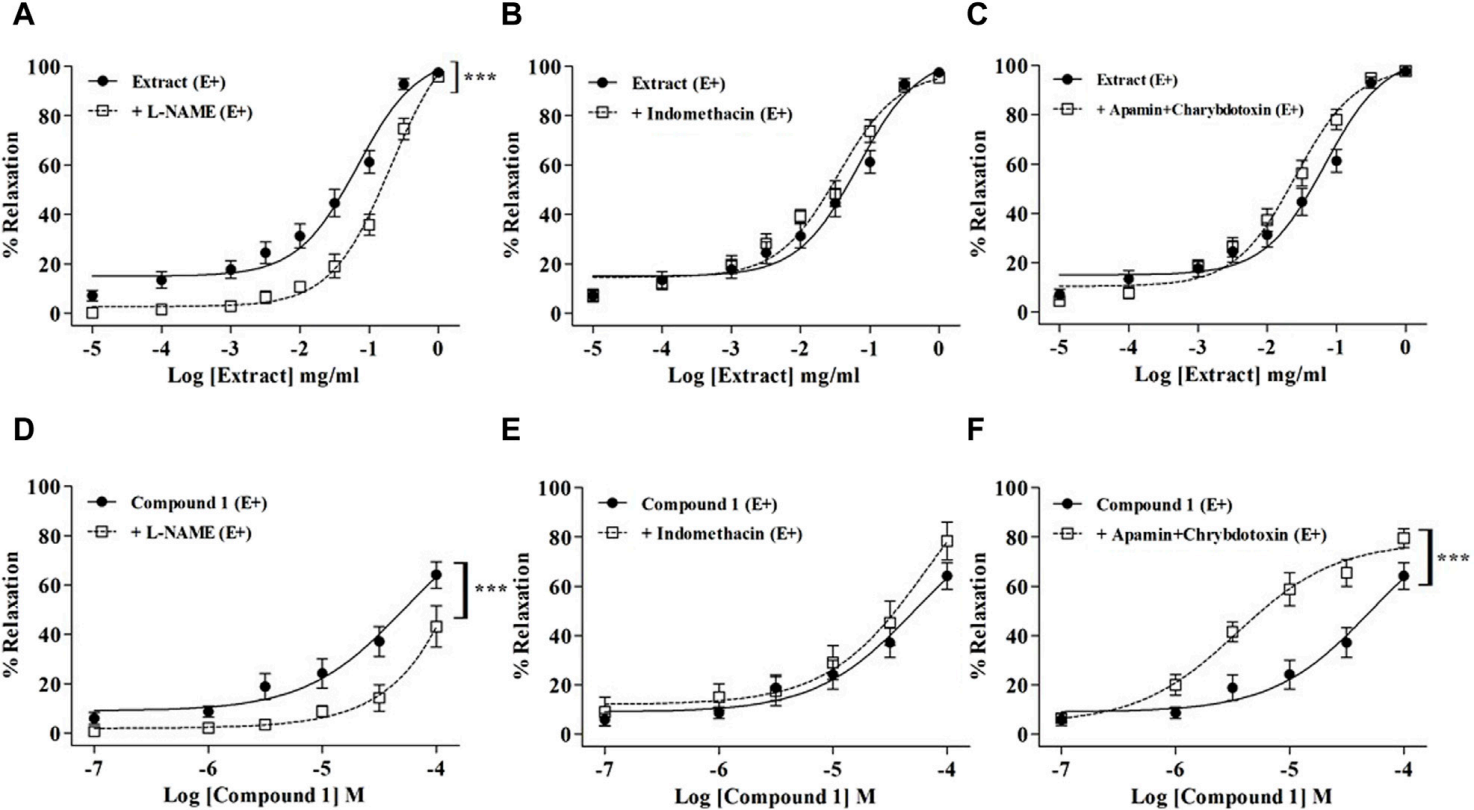
Relaxations of WL extract or quercetin 3-methyl ether 3′-O-*β*-xylopyranoside **(1)** on endothelium-intact (E+) MA rings pre-contracted with PE and pretreated with inhibitors of various endothelial signaling pathways including 10^−4^ M L-NAME **(A, D)**, 10^−5^ M indomethacin **(B, E)**, or 10^−7^ M apamin plus 10^−7^ M charybdotoxin **(C, F)**. The values are presented as the means ± SEM (n = 7–11); ^***^ *p* < 0.001.

L-NAME significantly reduced the relaxation induced by WL extract (Figure 2A) and compound **1** (Figure 2D) (*p* < 0.001), whereas indomethacin had no effect (Figures 2B,E). The incubation of mesenteric arteries with apamin and charybdotoxin did not modify the WL extract-induced relaxation (Figure 2C), while it enhanced the compound **1-**induced relaxation (Figure 2F) (*p* < 0.001).

### 3.3 The WL extract and its main compound increased the cGMP pathway

**FIGURE 3:**
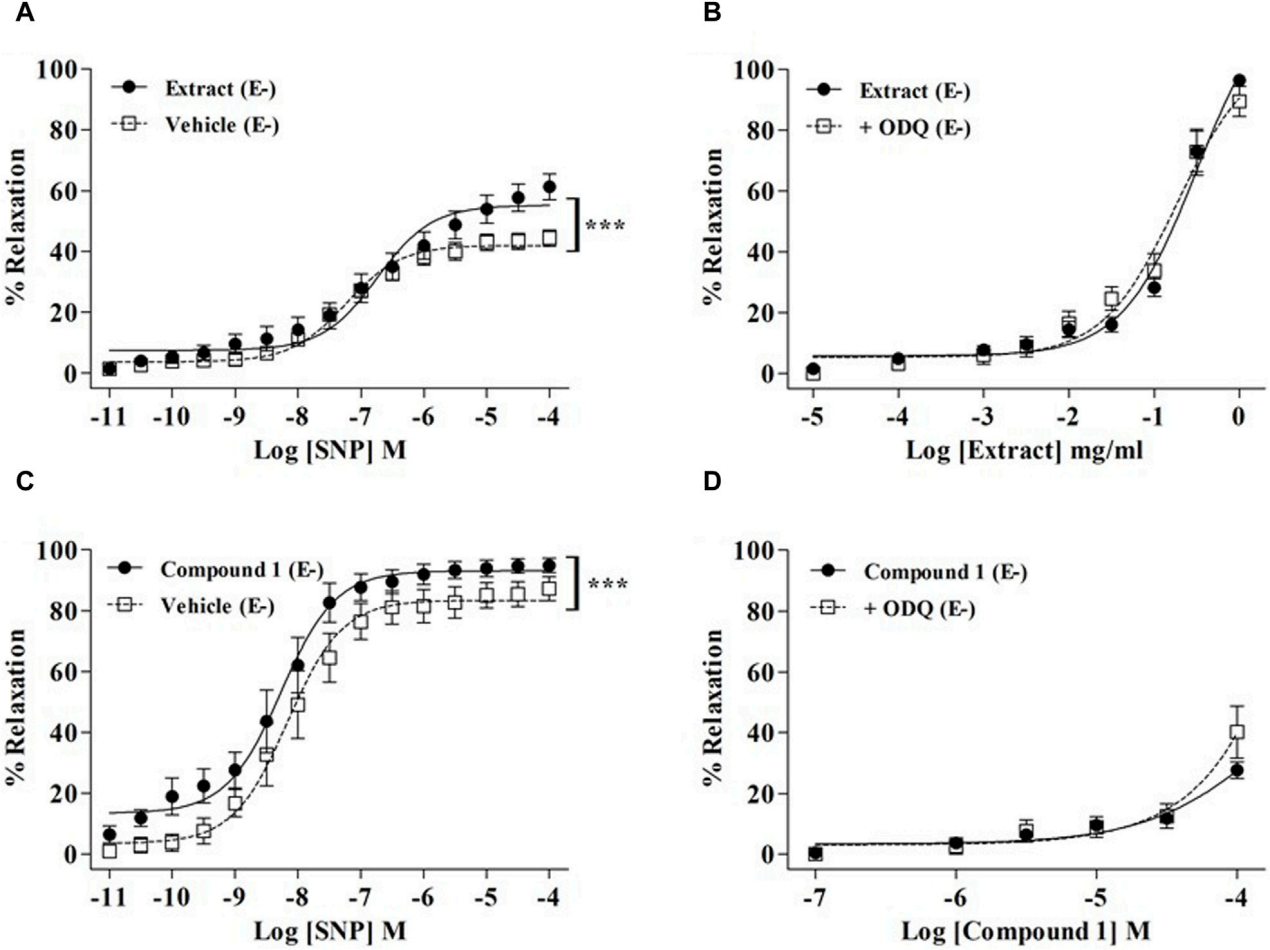
Effect of WL extract or quercetin 3-methyl ether 3′-O-*β*-xylopyranoside **(1)** on the sGC/CMP pathway. Endothelium-denuded (E−) MA rings were pretreated with WL extract, compound **1**, or vehicle and then pre-contracted with a 100 mM KCl solution **(A)** or PE (10^−5^ M) **(C)** prior to obtaining cumulative concentration–response for SNP (10^−11^–10^−4^ M). WL extract- **(B)** or compound **1 (D)**-induced relaxation on endothelium-denuded (E−) MA rings pre-contracted with PE (10^−5^ M) and ODQ (10–5 M). The values are presented as the means ± SEM (n = 7–11); ^***^ *p* < 0.001.

As endothelial NO production is strongly involved in WL extract- and compound-1-induced relaxations, we further explore whether WL extract or compound 1 might modulate signaling pathways downstream of NO in smooth muscle cells (VSMCs). As shown in Figures 3A,C, the NO donor SNP-induced relaxation was significantly enhanced in the presence of WL extract and compound **1** (*p* < 0.001). These data indicated that WL extract and compound **1** induced vasorelaxation through direct activation of sGC and/or by a downstream modulation of cGMP. To confirm whether a direct activation of sGC might be involved, the effect of ODQ was investigated. We found that ODQ did not modify the WL extract- or compound **1**-induced relaxations (Figures 3B,D).

### 3.4 The endothelium-independent effect was not reduced by K^+^ channel blockers

**FIGURE 4:**
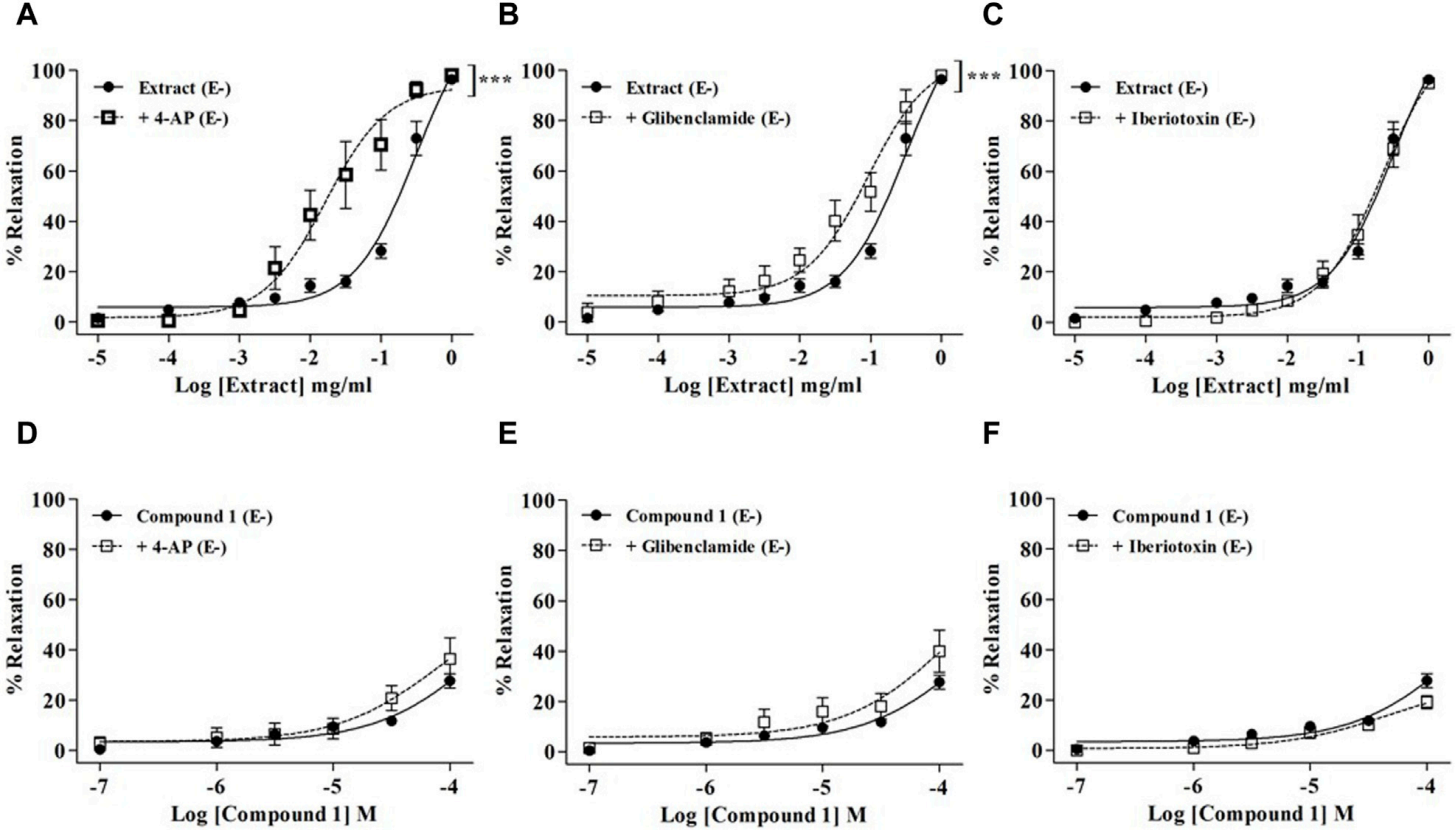
Relaxations of WL extract or quercetin 3-methyl ether 3′-O-*β*-xylopyranoside **(1)** on endothelium-denuded (E−) MA rings pre-contracted with PE (10^−5^ M) and pretreated with various K^+^ channel inhibitors including 10^−3^ M 4-AP **(A, D)**, 10^−5^ M glibenclamide **(B, E)**, or 10^−7^ M iberiotoxin **(C, F)**. The values are presented as the means ± SEM (n = 6–10); ^***^ *p* < 0.001.

As shown in Figure 4, none of the K^+^ channel blockers were able to reduce the relaxation induced by the WL extract or compound **1**. On the contrary, WL extract-induced relaxation was significantly enhanced in the presence of 4-AP and glibenclamide (Figures 4A,B).

### 3.5 The vasorelaxant effect of WL extract and compound 1 relied on receptor-operated Ca^2+^ channel inhibition

**FIGURE 5:**
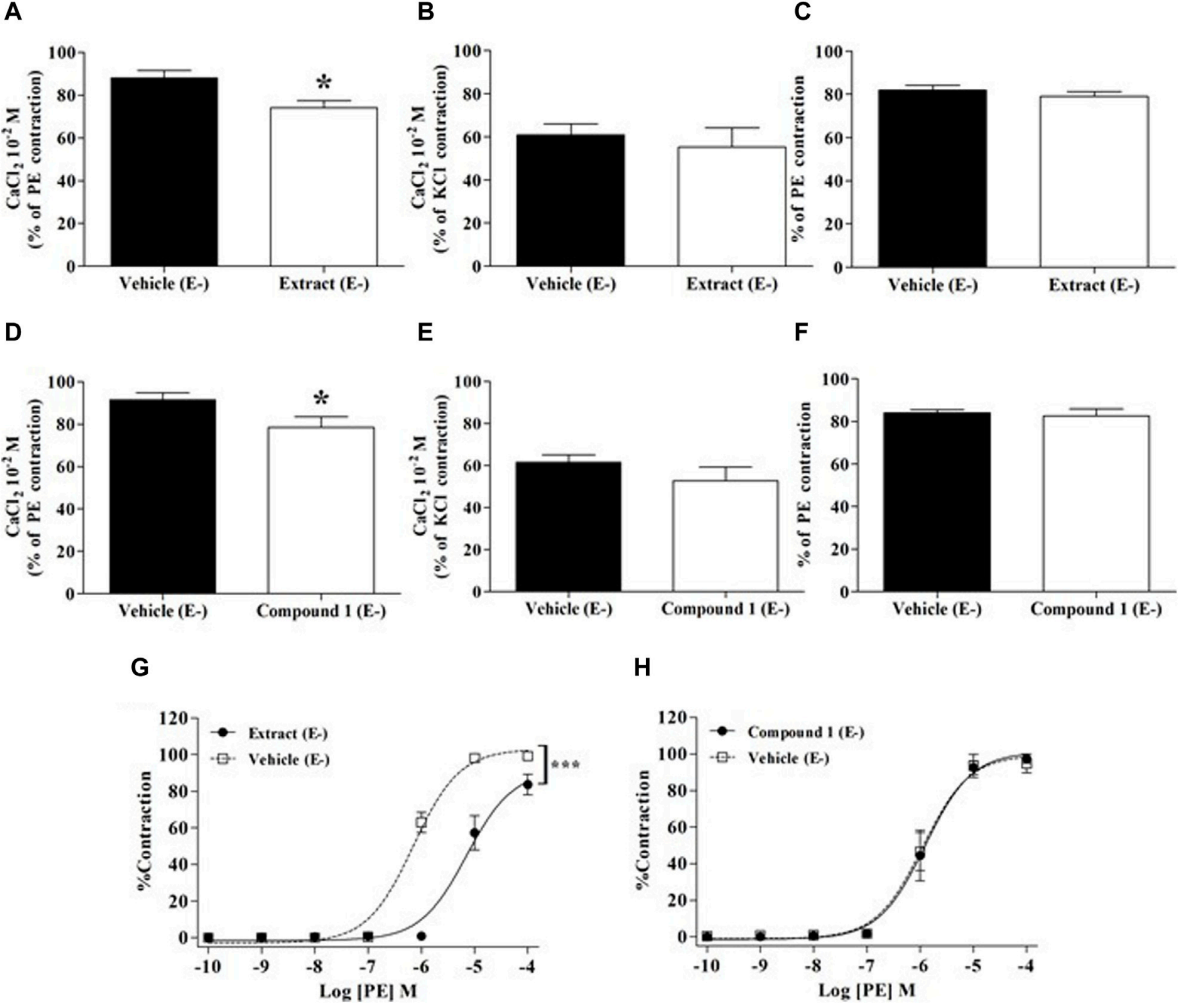
Effect of WL extract or quercetin 3-methyl ether 3′-O-*β*-xylopyranoside **(1)** on extracellular/intracellular Ca^2+^ fluxes and α1-adrenergic receptors. Experiments were performed in endothelium-denuded (E−) MA rings. CaCl_2_ (10^−2^ M) was added in the presence of vehicle, WL extract, or compound 1 in Ca^2+^-free Krebs solution, after pre-constriction with PE (10^−5^ M) **(A, D)** or 80 mM KCl **(B, E)**. In separate experiments, rings were pre-incubated with verapamil (10^−7^ M) for 30 min, then pre-incubated with vehicle, WL extract, or compound **1** before adding PE (10^−5^ M) **(C, F)**. Finally, rings were pre-incubated with WL extract, compound **1,** or vehicle, followed by contraction with cumulative concentrations of PE (10^−10^–10^−4^ M) **(G, H)**. The values are presented as the means ± SEM (n = 7–10); ^*^ *p* < 0.05 and ^***^ *p* < 0.001.

The contributions of extracellular or intracellular Ca^2+^ fluxes are presented in Figure 5. As compared to the vehicle, WL extract and compound 1 slightly but significantly reduced the contraction elicited by extracellular Ca^2+^ influx in PE-exposed rings (opening of receptor-operated Ca^2+^ channels: ROCCs, *p* < 0.05, Figures 5A,D), whereas they did not change either the extracellular Ca^2+^ influx in high KCl-exposed rings (opening of voltage-operated Ca^2+^ channel: VOCC) or the intracellular Ca^2+^ release from the SR (Figures 5B,C,E,F).

### 3.6 The WL extract blocked α_1_ receptor

As shown in Figure 5G, WL extract inhibited PE-induced contraction, suggesting an α1 receptor antagonism activity (*p* < 0.001), whereas compound **1** had no effect (Figure 5H).

### 3.7 WL extract and its main compound reduced blood pressure

**FIGURE 6:**
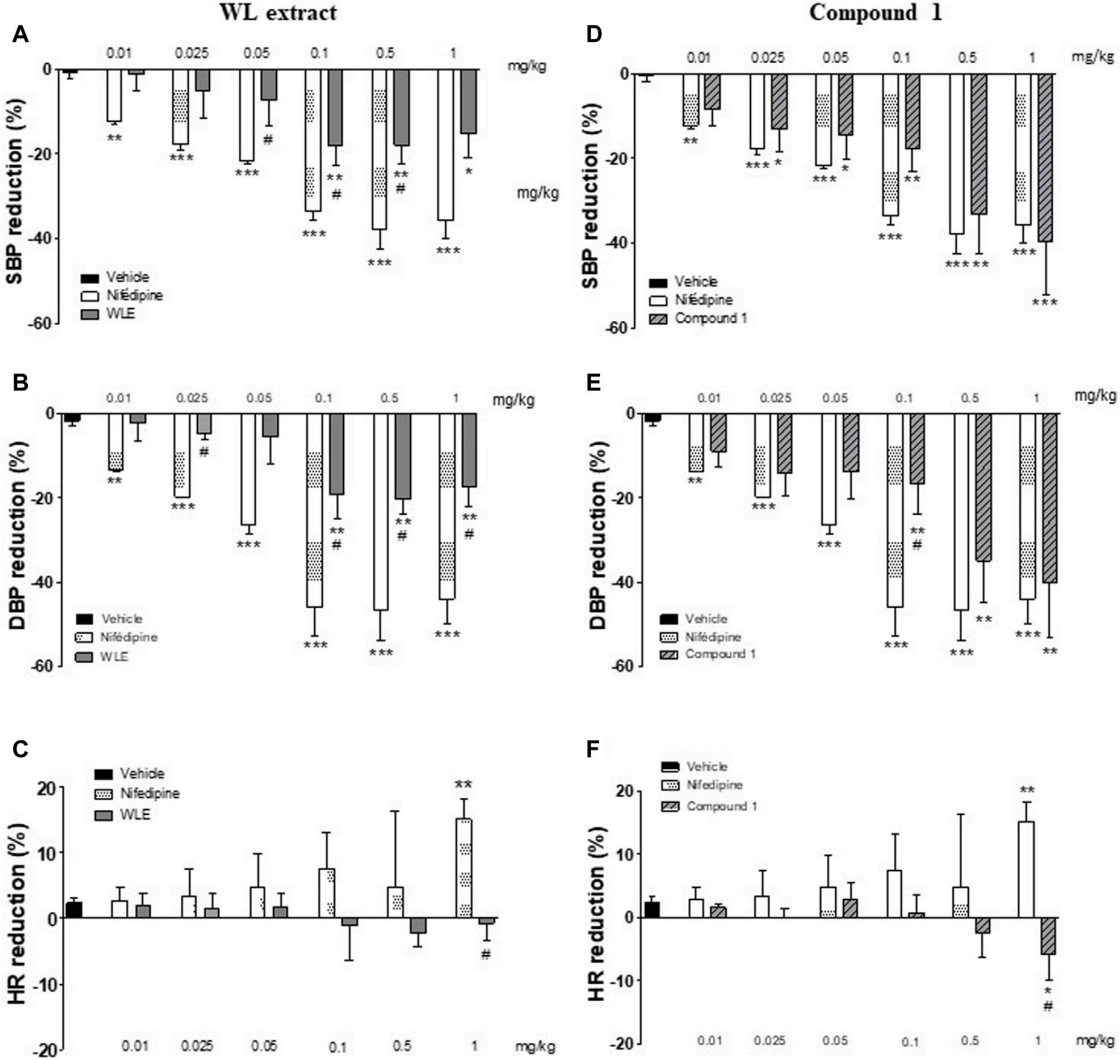
Acute hypotensive effect of WL extract and its main compound **1** (quercetin 3-methyl ether 3′-O-β-xylopyranoside) in rats. Percentage of reduction in SBP: systolic blood pressure **(A, D)**, DBP: diastolic blood pressure **(B, E)**, and HR: heart rate **(C, F)**. The values are presented as the means ± SEM (n = 4–5 rats per group). *p < 0.05, **p < 0.01, ***p < 0.001 vs. vehicle, and #*p* < 0.05 vs. nifedipine at the same dose.

As compared to the vehicle, the infusion of WL extract significantly decreased SBP and DBP at doses ranging from 0.025 to 1 mg/kg (Figures 6A,B). These hypotensive effects were lower compared to those of nifedipine. Interestingly, the effect of compound **1** in reducing blood pressure was similar to that of nifedipine (Figures 6D,E). Moreover, the rebound tachycardia found at the highest dose of nifedipine was not shown with WL extract or compound **1** (*p* < 0.05, Figures 6C,F).

## 4 Discussion

The new findings of our study are that 1) an ethanolic extract of *N*. *pubescens* petals induced a vasorelaxant effect on resistance vessels through mechanisms combining endothelium-dependent and -independent pathways; 2) this direct vascular effect translated into an acute hypotensive effect *in vivo*; and 3) the main component of the extract, quercetin 3-methyl ether 3′-O-*β*-xylopyranoside, induced vasorelaxant effects that can, at least partly, explain the relaxing actions of the extract.

In line with the traditional uses of extracts from *Nymphaea* petals to promote erectile function through an increase in endothelial NO production (Mireille et al., 2016), the present study showed that the *N*. *pubescens* extract induced a potent vasorelaxant effect relying on endothelium-dependent mechanisms. However, the relaxant effect of the extract was not abolished by endothelium removal, as illustrated by the switch to the right of the relaxation curves without a decrease in the E_max_ value of the extract, thus indicating that endothelium-independent mechanisms are also important contributors. Our data demonstrated that the effects of the extract involved the activation of the NO-GCs-cGMP pathway. Indeed, the relaxant effect was inhibited by a NOS inhibitor, indicating the capacity of the extract to stimulate endothelial NO production, but the results revealed that the extract also promoted NO signaling at the level of VSMCs. Moreover, the extract enhanced the effect of the NO donor but did not act itself as a direct sGC activator. Altogether, these results suggest that the phosphodiesterase inhibitory properties exhibited by the extract (Panklai et al., 2023) may contribute to its relaxant effect, but the causal relationship has to be investigated in future studies. Regarding the effect of the extract on calcium fluxes, only extracellular calcium entry through ROCCs was weakly but significantly inhibited by the extract, without any effect on VOCC or intracellular calcium release from SR. In addition, the extract exhibited a weak inhibition of the PE-induced contracting effect, suggesting α_1_-receptor antagonism. Regarding this latter mechanism, as ROCCs are activated by agonists acting on G-protein-coupled receptors, such as phenylephrine, we cannot exclude that the inhibitory effect of the extract on ROCC contributes to, or is responsible for, the reduction of the PE constricting action. The relaxation of VSMCs is also mediated by hyperpolarization secondary to the opening of different types of K^+^ channels, including Kv, K_ATP_, and K_Ca_ (Bosnjak, 1993). Here, we showed that the effect of *N*. *pubescens* extract was not reduced by any of the K^+^ channel blockers. In contrast, 4-aminopyridine and glibenclamide enhanced extract-induced relaxation. These unexpected results could be explained by the interaction of several active molecules on different isoforms of these K^+^ channels, the specificity of which deserves further investigation.

The main compound of the extract is the quercetin derivative, quercetin 3-methyl ether 3′-O-*β*-xylopyranoside, with a content of 1.73% (w/w) (Panklai et al., 2022). The present study reported for the first time that this quercetin derivative had a vasodilatory effect, albeit not very potent, as illustrated by its E_max_, which did not reach 100% relaxation. These data suggest that this compound is probably involved in the relaxant effect of the extract, but other compounds that remain to be identified are also involved. Of interest, our previous work demonstrated that the WL extract also contains, to a lesser extent, quercetin 3′-O-β-xylopyranoside, quercetin, 3-O-methylquercetin, kaempferol, and 3-O-methylkaempferol (Panklai et al., 2023). Mechanistically, the main compound quercetin 3-methyl ether 3′-O-*β*-xylopyranoside shared with the extract the same effects on the NO-sGC-cGMP pathway and ROCCs but did not present an α_1_-receptor antagonistic effect. As compared to quercetin, which is a known vasodilator of MA (Nishida and Satoh, 2013; Satoh and Nishida, 2014), our data indicate that the methyl ester group in the 3′ position and the presence of the xylopyranoside derivative did not alter the vasorelaxant properties. Nevertheless, a few mechanistic differences exist, as the relaxing effects of quercetin on MA were mainly related to EDHF and slightly through the endothelial NO pathway (Nishida and Satoh, 2013).

To determine whether the vasorelaxant effects observed *ex vivo* were still present after *in vivo* administration, the acute hypotensive effects of WL extract and quercetin 3-methyl ether 3′-O-*β*-xylopyranoside were investigated. The results showed that the extract acutely reduced blood pressure, with an effect that was, however, lower than that of nifedipine, used as a comparator. The limitation of our experiment is that it was conducted in normotensive rats, i.e., rats with a normal endothelial function, thus probably hampering the endothelial component of the relaxant effect of the WL extract. Further studies exploring the effect of the extract on animal models of hypertension are now required. However, this hypotensive action is in agreement with the anti-hypertensive effect previously reported with an aqueous extract from the flowers of another *Nymphaea (lotus)* L. (Kameni et al., 2019). The hypotensive effect of quercetin 3-methyl ether 3′-O-*β*-xylopyranoside was found to be similar to that of nifedipine. Again, these data indicate that the methyl ester group in the 3′position and the presence of the xylopyranoside derivative did not reduce the hypotensive effect of quercetin (Duarte et al., 2001; Brüll et al., 2015). It is noteworthy that the *in vivo* hypotensive effect of quercetin 3-methyl ether 3′-O-*β*-xylopyranoside is more pronounced than the effect of the WL extract, while its efficacy to induce mesenteric artery relaxation is less. The pharmacokinetic parameters of compound **1** deserve to be further explored to understand this potent hypotensive activity. Moreover, as compared to nifedipine, this quercetin derivative did not induce rebound tachycardia at high concentrations but, on the contrary, reduced heart rate. Altogether, these results suggest that a direct cardiac effect is possible with this compound. Further studies will be needed to better characterize this putative mechanism.

## 5 Conclusion

The present study showed that the ethanolic extract of *N. pubescens* petals induced vascular relaxing effects on the rat mesenteric artery, relying not only on the potentiation of NO production by the endothelium but also on endothelium-independent mechanisms such as the activation of the sGC-cGMP pathway and inhibition of ROOCs. Our data provide pharmacological evidence for the traditional use of this plant in diseases associated with hampered endothelial function. The main compound, quercetin 3-methyl ether 3′-O-*β*-xylopyranoside, is a possible contributor to these effects, but other molecules are likely involved that remain to be identified.

## Data Availability

The original contributions presented in the study are included in the article/Supplementary Material; further inquiries can be directed to the corresponding author.

## Abbreviations

4-AP, 4-aminopyridine; ACh, acetylcholine; cGMP, cyclic guanosine monophosphate; COX, cyclooxygenase; DBP, diastolic blood pressure; DMSO, dimethyl sulfoxide; E, endothelium; EDHF, endothelium-derived hyperpolarizing factor; EGTA, ethylene glycol-bis (2-aminoethylether)-N,N,N′,N′-tetraacetic acid; HR, heart rate; IP_3_, inositol 1,4,5 trisphosphate; L-NAME, N^G^-nitro-L-arginine methyl ester; MA, mesenteric artery; NO, nitric oxide; NOS, nitric oxide synthase; ODQ, 1H-[1,2,4]oxadiazolo[4,3-a]quinoxalin-1-one; PE, phenylephrine; ROCCs, receptor-operated Ca^2+^ channels; SBP, systolic arterial blood pressure; sGC, soluble guanylyl cyclase; SNP, sodium nitroprusside; SR, sarcoplasmic reticulum; VOCCs, voltage-operated Ca^2+^ channels; WL, water lily.
